# Distinct Reward Properties are Encoded via Corticostriatal Interactions

**DOI:** 10.1038/srep20093

**Published:** 2016-02-02

**Authors:** David V. Smith, Anastasia E. Rigney, Mauricio R. Delgado

**Affiliations:** 1 Department of Psychology, Rutgers University, Newark, NJ 07102, USA

## Abstract

The striatum serves as a critical brain region for reward processing. Yet, understanding the link between striatum and reward presents a challenge because rewards are composed of multiple properties. Notably, affective properties modulate emotion while informative properties help obtain future rewards. We approached this problem by emphasizing affective and informative reward properties within two independent guessing games. We found that both reward properties evoked activation within the nucleus accumbens, a subregion of the striatum. Striatal responses to informative, but not affective, reward properties predicted subsequent utilization of information for obtaining monetary reward. We hypothesized that activation of the striatum may be necessary but not sufficient to encode distinct reward properties. To investigate this possibility, we examined whether affective and informative reward properties were differentially encoded in corticostriatal interactions. Strikingly, we found that the striatum exhibited dissociable connectivity patterns with the ventrolateral prefrontal cortex, with increasing connectivity for affective reward properties and decreasing connectivity for informative reward properties. Our results demonstrate that affective and informative reward properties are encoded via corticostriatal interactions. These findings highlight how corticostriatal systems contribute to reward processing, potentially advancing models linking striatal activation to behavior.

A vast array of behaviors can be shaped by rewards^1^,^2^. Over the past few decades, this observation has sparked a myriad of studies highlighting the role of various brain regions—including the prefrontal cortex^3^, midbrain^4^, and striatum^5^—in reward processing. While many recent efforts focus on the comparison of different types of rewards^6^, few studies have compared the intrinsic properties of reward. Indeed, the mere receipt of any type of reward carries multiple signals that modulate our affect (e.g., how we feel after a positive outcome) while simultaneously providing information that guides our future behavior (e.g., this action was correct and led to the desirable outcome).

Studies that separately focus on either affective (e.g., see ref. 5) or informative (e.g., see ref. 7) properties find similar effects, including increased activation within the striatum. The observation that both affective and informative reward properties, when studied separately, produce similar effects creates considerable ambiguity for understanding the reward response. This ambiguity has important implications for clinical and social perspectives. The idea that multiple reward properties are imbedded within the observed neural response hinders models linking reward-processing deficits to different psychopathologies^8^. For example, deficits in affective processing of a reward^9^ can influence extraction of information during performance feedback^10^. Moreover, a variety of political and educational institutions rely on unambiguous models of reward processing in order to employ effective incentive mechanisms^11^. Taken together, these issues highlight the importance of parsing reward into distinct properties.

Yet, efforts to parse reward into distinct properties have been met by two significant challenges. First, affective and informative reward properties are difficult to separate using existing procedures. Although recent work demonstrates that information^12^ and affective stimuli^13^ can individually increase activation in the striatum, less is known about the computations that encode each reward property. Second, the activation of a single brain region (e.g., striatum) may be necessary but not sufficient to encode distinct reward properties. Disentangling reward properties may therefore require characterizing how other brain regions interact with the striatum during reward processing^14^. Indeed, recent work has utilized this approach to ascribe multiple functions to a single region depending on its connectivity with other brain regions^15^,^16^. Taken together, these issues have limited our understanding of how distinct reward properties contribute to reward processing signals observed in the striatum.

We addressed these challenges by developing a procedure that emphasized affective and informative properties of reward in separate card-guessing tasks (Fig. 1). Both card tasks employed an identical trial structure—drawing a card from one of three decks—but the cards received in each task had distinct influences on the principal reward in the task attained at the end of the experiment (i.e., monetary compensation). Specifically, the Affective Card Task (ACT; Fig. 1A) emphasized the acquisition of *points* that would permit entry into a bonus game for monetary rewards at the end of the experiment. The Informative Card Task (ICT; Fig. 1B) emphasized the acquisition of *information*, which would assist with successful performance in the bonus game. Crucially, the bonus task structure helps participants focus on the distinct goals of each task separately (i.e., get points or get information) while equating the importance of both tasks with respect to earning the monetary reward (i.e., need points to play the bonus game; need information to do well and earn money). Our analyses focused on two key questions. First, does the striatum encode responses to both types of reward properties? Second, does the strength of connectivity with striatum distinguish distinct reward properties?

## Results

### Behavioral Effects of Affective and Informative Reward Properties

Our behavioral analyses focused on supporting the affective and informative distinctions across our tasks. We evaluated whether affect was differentially emphasized in the ACT and ICT by comparing several affective responses to the tasks. Next, we evaluated whether information was differentially emphasized in the ACT and ICT by examining responses to feedback. Both tasks delivered feedback that varied in magnitude: points in the ACT and information in the ICT (see Supplemental Methods).

#### Emphasizing Affect

We tested whether our tasks differed in terms of evoked affect using multiple measures. First, we used the preference task, which measured individual differences in subjective value for affect and information. We found that participants significantly valued affective feedback cues over informative feedback cues [*M*_info_ = 39.34%; t_(32)_ = −3.37, p = 0.002, one-sample t-test against indifference (50%)]. Second, we examined post-scan self-report measures assessing subjective enjoyment, motivation, and excitement for each task (Likert-scale: 1–7). Although participants’ self-reported enjoyment was similar for the ACT and ICT (*t*_(32)_ = 1.18, *p* = 0.25), we found that a significant proportion of participants (21 out of 33) expressed more enjoyment for the ACT compared to the ICT (*χ*^*2*^ = 13.82, *p* = 0.001), suggesting that the ACT induced more affect (in terms of subjective pleasure) than the ICT. In addition, other self-report measures were comparable, both in terms of inter-participant distributions (excitement: *χ*^*2*^ = 3.45, *p* = 0.18; motivation: *χ*^*2*^ = 1.27, *p* = 0.52) and average differences in Likert-scale responses (excitement: *t*_(32)_ = 1.64, *p* = 0.11; motivation: *t*_(32)_ = 0.88, *p* = 0.39). We also found that participants who earned more points in the ACT tended to prefer the ACT over the ICT at a trend level (*r*_(31)_ = 0.30, *p* = 0.08) and express greater enjoyment for the ACT (rank-order correlation: ρ = 0.48, *p* = 0.004), suggesting that evoked affect (and hence enjoyment) in the ACT was linked to points received. Finally, in a subset of participants with usable GSR data, we examined whether feedback delivered in the ACT evoked greater skin conductance responses than feedback delivered in the ICT (see Supplemental Methods). Our results suggested that feedback-dependent responses were marginally significantly higher during the ACT relative to the ICT (*t*_(13)_ = 1.68, *p* = 0.058, one tailed). We deem these results exploratory because of the limited amount of usable GSR data collected during scanning. Yet, these observations, when considered with our other affective measures, suggest that our procedures successfully emphasized affect within the ACT.

#### Emphasizing Information

**Figure 1:**
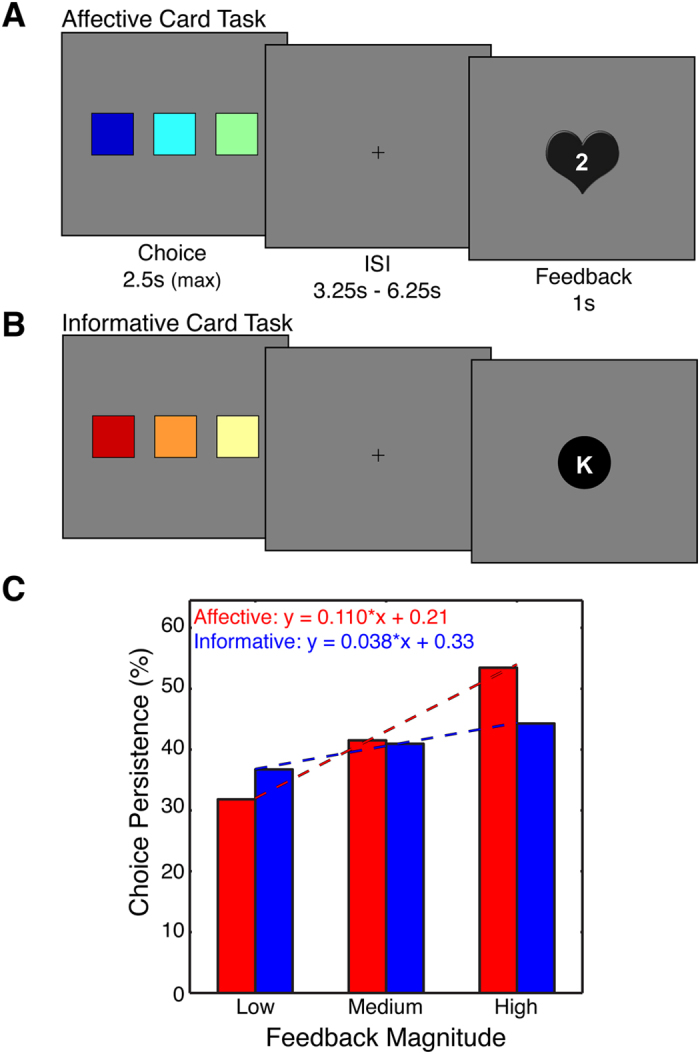
Experimental Tasks and Choice Behavior. Affective and informative components of reward were investigated using two parallel card-guessing tasks. Both card-guessing tasks were predicated on distinct goals related to a bonus game played for monetary compensation at conclusion of the experiment. (**A**) In the Affective Card Task (ACT), the goal for the participants was to earn enough points to play in the bonus game. On each trial of the ACT, participants chose from three decks of cards containing variable amounts of points (1–3). (**B**) In the Informative Card Task (ICT), the goal for the participants was to learn the contents of each deck of cards because the bonus game would explicitly test this knowledge through a series of questions asking the participant which deck was most likely to contain a shown letter. To ensure valid comparisons with the ACT, the ICT utilized an identical trial structure, where participants chose between three decks containing letters (D, K, X) that appeared with different probabilities in each deck (50%, 33%, 17%). (**C**) To facilitate comparisons across tasks, both tasks delivered no feedback on a subset of trials (i.e., no points or no letter). Thus, feedback magnitude in both tasks was anchored to a common minimum, allowing us to make meaningful comparisons across tasks. Our behavioral analysis indicated that choice persistence—the likelihood of staying with a particular deck choice—increased with increasing feedback, an effect that was more pronounced during the ACT. Shown are the best-fit lines. We note that slopes of these lines were variable across participants (affective: range = −0.21:0.44, SD = 0.15; informative: range = −0.22:0.34, SD = 0.10), suggesting that choice persistence in some participants was not influenced by feedback magnitude.

The key informational construct within our procedures relates to learning the association between the letters and the decks in the ICT in order to answer questions correctly—and thus earn money—in the bonus task. Participants were generally successful at acquiring the relevant information and answering questions correctly in our bonus task (*M* = 69.36%; *SE* = 3.45%). No other information will help answer questions correctly—and hence earn money—in our bonus task. Nevertheless, we note that other types of information may be present in the ACT if the outcomes (i.e., points) predicted subsequent outcomes. We therefore assessed the likelihood of persisting with a given choice for each level of feedback (excluding the no feedback condition; see Supplemental Methods). In both tasks, we found that participants were more likely to stick with a choice with greater feedback magnitude (Fig. 1C; *F*_(2,64)_ = 15.39, *p* < 0.0001, ε = 0.79), an effect that interacted with the type of feedback (*F*_(2,64)_ = 3.33, *p* = 0.045, ε = 0.95). In addition, the effects of choice persistence, as represented by the slope of the line between low and high feedback, was highly variable across participants (affective: range = −0.21:0.44, SD = 0.15; informative: range = −0.22:0.34, SD = 0.10), indicating that some participants were not influenced by feedback magnitude. Although the effects of choice persistence could suggest that information guided choice in the ACT, it is important to recognize that collecting additional points in the ACT would only permit entry into the bonus game and would not provide useful information for earning money (i.e., answering questions correctly). In addition, while our key analyses controlled for response time differences, we note that the ACT (*M* = 965 ms) and the ICT (*M* = 984 ms) did not differ according to response time (*t*_(32)_ = −0.76, *p* = 0.45).

### Striatal Responses to Affective and Informative Reward Properties

**Figure 2:**
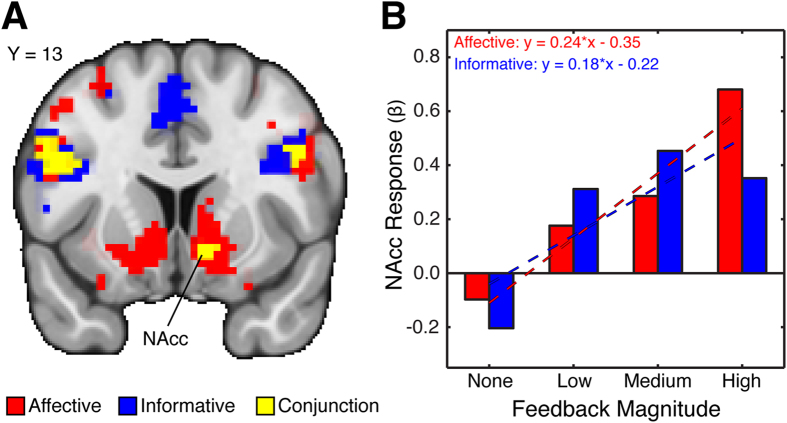
Affective and Informative Reward Properties Evoke Similar Responses within the Nucleus Accumbens. (**A**) To identify brain regions whose activation tracked increasing levels of affective and informative reward properties, we constructed a parametric model based on normalized feedback magnitude. We found that affective (red) and informative (blue) feedback evoked activation in the striatum. To identify regions responding to both affective and informative feedback, we conducted a cluster-based conjunction analysis using the minimum statistic, which identified a cluster within nucleus accumbens (yellow) [MNI_x,y,z_ = 9, 14, −7 (26 voxels)]. All areas of activation passed an initial cluster-forming threshold of *z* = 3.1, with whole-brain cluster correction at *p* = 0.05 (corrected for multiple comparisons). (**B**) Interrogation of the ventral striatum region revealed linear trends of activation for both conditions, with higher activation corresponding to higher feedback magnitude and lower activation corresponding to lower feedback magnitude. For descriptive purposes, the slopes of the best-fit lines illustrate the similarity in response profiles across increasing affective and informative feedback magnitude.

Although multiple studies demonstrate that the striatum responds to rewarding stimuli^17^,^18^, it remains unclear how these signals are computed for distinct reward properties. To investigate this issue, we identified brain regions whose activation tracked trial-to-trial fluctuations in feedback magnitude in both card tasks. Our analysis indicated that increasing feedback magnitude was encoded by several regions for affective (Table 1; Fig. 2B) and informative (Table 2; Fig. 2B) reward properties. Next, we performed a cluster-based conjunction analysis using the minimum cluster statistic^19^ to identify regions exhibiting similar responses to affective and informative reward properties. This analysis revealed multiple regions (Table 3), including a cluster in the right nucleus accumbens (NAcc) (Fig. 2A) that encoded feedback magnitude for both affective and informative reward properties (Fig. 2B).

We probed the functional significance of NAcc responses to affective and informative reward properties by examining their relationship with behavior. First, we tested whether responses to informative reward properties predicted if participants successfully utilized information in the post-scan bonus game—a key test of our of NAcc results. Crucially, our analysis indicated that greater responses to informative reward properties predicted improved performance in the post-scan bonus game (*r*_(31)_ = 0.4538, *p* = 0.008). This relationship was not observed for responses to affective reward properties (*r*_(31)_ = −0.21, *p* = 0.24). Next, although we do not have an analogous behavioral or physiological variable to test the functional significance of NAcc responses to affective reward properties, we conjectured that affective responses might be inversely related to individual differences in subjective value for information. Nevertheless, we did not find a relationship between subjective value for information and affective responses (*r*_(31)_ = 0.1756, *p* = 0.3283) or even informative responses (*r*_(31)_ = 0.0330, *p* = 0.8551). Finally, we tested whether affective responses were correlated with behavioral metrics tied to putative information utilization within the ACT itself: a) proportion of low-value deck choice and b) choice persistence. None of these metrics correlated with NAcc responses to affective reward properties (all *P*s > 0.5455), suggesting that the ACT was not contaminated by meaningful information.

**Figure 3:**
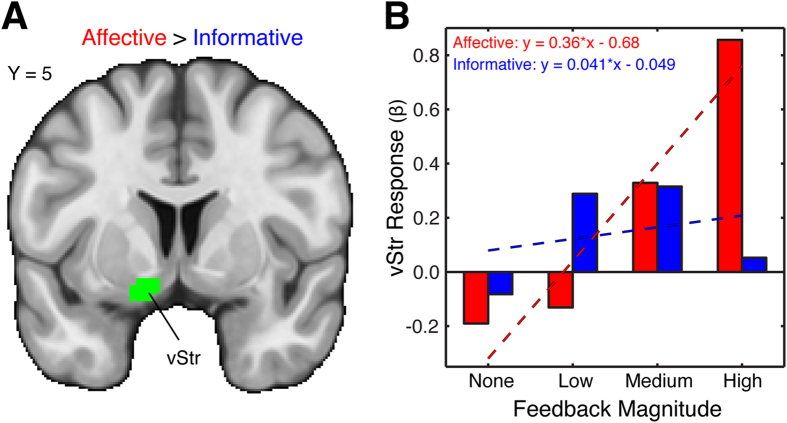
Affective Reward Properties Evoke Greater Responses within the Ventral Striatum. (**A**) We also contrasted responses to affective and informative reward properties. This analysis revealed a cluster within ventral striatum [MNI_x,y,z_ = −12, 5, −13 (22 voxels)] that responded more to affective reward properties compared to informative reward properties. This area of activation passed an initial cluster-forming threshold of *z* = 3.1, with whole-brain cluster correction at *p* = 0.05 (corrected for multiple comparisons). (**B**) Interrogation of the ventral striatum region revealed a linear trend of activation for the affective condition, with higher activation corresponding to higher feedback magnitude and lower activation corresponding to lower feedback magnitude. For descriptive purposes, the slopes of the best-fit lines illustrate the difference in response profiles across increasing affective and informative feedback magnitude.

We also directly contrasted the affective and informative responses to identify regions whose activation changes according to reward property. Although no regions exhibited greater responses to informative reward properties relative to affective reward properties, we found three regions that were more sensitive to affective reward properties. These regions included the middle frontal gyrus (MNI_x,y,z_ = −39, 26, 47; 26 voxels, *p* = 0.0133), the paracingulate gyrus (MNI_x,y,z_ = 9, 53, −4; 22 voxels, *p* = 0.0307), and the left ventral striatum (vStr) (MNI_x,y,z_ = −12, 5, −13; 22 voxels, *p* = 0.0307). These regions encoded feedback magnitude for affective reward properties but not informative reward properties; we focus on the left vStr cluster in Figure 3 because of our hypotheses regarding the striatum. We note that the contrast effect in left vStr was also uncorrelated with all of our behavioral metrics (all *P*s > 0.3641), suggesting that behavioral differences did not contribute to neural differences.

Our results are potentially suggestive of lateralization within the striatum. To examine this possibility, we conducted a *post hoc* analysis comparing responses in left vStr from the direct contrast analysis and right NAcc from the conjunction analysis to their mirrored counterpart regions in the contralateral hemisphere. We found that responses within the mirrored regions were highly correlated with both our contrast findings for the vStr (*r*_(31)_ = 0.61, *p* < 0.001) and our main conjunction findings for the NAcc (affective: *r*_(31)_ = 0.71, *p* < 0.001; informative: *r*_(31)_ = 0.56, *p* < 0.001). In addition, responses within the mirrored regions did not differ from our contrast findings within vStr (*t*_(32)_ = 0.78, *p* = 0.44) or our main conjunction findings within NAcc (affective: *t*_(32)_ = −0.78, *p* = 0.44; informative: *t*_(32)_ = 1.87, *p* = 0.07). Taken together, these results do not support the idea that striatal responses in our study are lateralized, which is consistent with prior reports^20^.

### Corticostriatal Interactions Distinguish Affective and Informative Reward Properties

Our results suggest a general role for the NAcc in processing affective and informative reward properties. Yet, these computations may depend on interactions with other brain regions—an observation that would highlight independent pathways for distinct reward properties. Indeed, if differences between reward properties are not reflected in NAcc changes, then perhaps they are expressed in connectivity strength between other regions and NAcc. We tested this idea using a psychophysiological interaction (PPI) analysis with the right NAcc defined by the conjunction analysis as our seed region^21^ (Fig. 2A). This analysis allowed us to identify regions whose connectivity with NAcc increases as a function of increasing feedback magnitude. We then contrasted feedback-dependent changes in connectivity in the affective and informative conditions to reveal regions whose connectivity with NAcc increases depending on reward property. Our analysis did not reveal any regions exhibiting differential connectivity with NAcc. We obtained similar results when using left vStr region (Fig. 3A) as our seed region.

**Figure 4:**
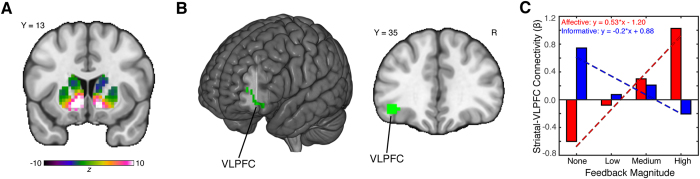
Corticostriatal Interactions Distinguish Affective and Informative Reward Properties. We utilized a psychophysiological interaction (PPI) analysis to test whether the magnitude of affective and informative reward properties influenced functional connectivity with the striatum (defined by the union of the caudate, putamen, and nucleus accumbens within the Harvard-Oxford atlas). (**A**) We used independent component analysis to obtain a set of striatal networks—spatial maps containing distinct patterns of responses. Using a variant of dual-regression analysis^16^,^24^, we found that one striatal network (i.e., independent component) that responded to affective and informative reward properties (shown as a map of *z*-scores). (For clarity, other independent components are not shown.) We used the task-sensitive striatal network as the “seed” region in our PPI analysis; its temporal dynamics were extracted from each participant using a variant of the dual-regression analysis. (**B**) Our task-sensitive striatal network exhibited dissociable patterns of functional connectivity with ventrolateral prefrontal cortex (VLPFC), with greater connectivity during affective feedback compared to informative feedback. We note that VLPFC activation passed an initial cluster-forming threshold of *z* = 2.3, with whole-brain cluster correction at *p* = 0.05 (corrected for multiple comparisons). (**C**) These corticostriatal interactions increased during affective feedback and decreased during informative feedback, suggesting that inputs from VLPFC may distinguish distinct reward properties. For descriptive purposes, the slopes of the best-fit lines illustrate the differences in connectivity strengths across increasing affective and informative feedback magnitude.

These observations suggest that corticostriatal interactions tied to affective and informative reward properties are not restricted to discrete regions of the striatum. Instead, the striatum, like other processing hubs^16^, may be organized into multiple independent networks reflecting its distributed, yet overlapping, anatomic^22^ and functional^23^ connections. Thus, to facilitate dissociation of overlapping signals within the striatum, we applied an analytical framework consisting of independent component analysis (ICA) combined with dual-regression analysis^16^,^24^. This approach allowed us to segregate the striatum into multiple overlapping networks. After adjusting for multiple comparisons (Bonferroni), only one striatal network responded to affective (*t*_(32)_ = 10.38, *p* < 0.001, corrected) and informative (*t*_(32)_ = 4.49, *p* < 0.001, corrected) reward properties (Fig. 4A). We then used this task-sensitive striatal network in another PPI analysis to test whether corticostriatal interactions distinguish affective and informative reward properties. Strikingly, we found one cluster in ventrolateral prefrontal cortex (VLPFC) (peak *z*-stat = 3.6 at MNI_x,y,z_ = −39, 35, −10; 49 voxels, *p* = 0.0411) that exhibited enhanced striatal connectivity for affective relative to informative reward properties (Fig. 4B). These corticostriatal interactions increased during affective feedback and decreased during informative feedback, suggesting that coupling between striatum and VLPFC may distinguish distinct reward properties (Fig. 4C). We note that these VLPFC results were robust to a modified PPI analysis that also included an additional PPI regressor for the presentation of feedback^25^. In addition, we also found that differences in connectivity were not due to differences in subjective value for information (*r*_(31)_ = 0.03, *p* = 0.85), suggesting that our results were not due to differences in preferences. Differences in choice persistence, however, could potentially contribute to differences in connectivity (*r*_(31)_ = −0.29, *p* = 0.10).

## Discussion

The complexity of reward has made it difficult to gain insight into the precise computations that underlie reward processing. Specifically, the ambiguity as to how affective and informative reward properties differentially drive brain activation and shape behavior hinders a range of potential applications—from incentive structures within our educational system^11^ to biological models of psychopathologies^8^. To address this problem, we developed a procedure that allowed us to independently examine affective and informative properties of reward. Our results indicated that affective and informative reward properties increase activation throughout much of the striatum, with two notable aspects: similar responses to both properties coded within the nucleus accumbens (NAcc) and increased affective responses in a posterior ventral striatum (vStr) subregion. Neither striatal subregion exhibited distinct patterns of connectivity with prefrontal cortex, potentially reflecting the hub-like nature of striatum^22^,^23^. To resolve this issue, we applied an analytical technique that parsed the striatum into multiple independent networks. One of these striatal networks responded to both reward properties and showed distinct patterns of connectivity with prefrontal cortex. Specifically, a region of ventrolateral prefrontal cortex (VLPFC) increased connectivity with the striatum during the receipt of affective reward properties and decreased connectivity with the striatum during the receipt of informative reward properties. Our results highlight how responses to distinct reward properties can be differentiated based on corticostriatal connectivity.

These findings build on a growing set of observations linking striatal activation to the prediction and receipt of rewarding stimuli. Our study extends previous work examining the interplay between reward and information^26^,^27^ by focusing on the distinct contributions of affect and information during the receipt of reward. Unfortunately, affective and informative reward properties are commonly conflated because the mere receipt of a reward modulates affect (e.g., increase pleasure) while simultaneously providing information (e.g., reinforcing behavior). We note that individual reward properties may not be completely separable, and we acknowledge that there is some degree of residual affect in the informative card task and some degree of residual information in the affective card task. Nevertheless, our procedures emphasized each property independently, permitting relative comparisons of affective and informative reward properties.

Unlike previous work, our experiment also highlights how corticostriatal interactions distinguish affective and informative reward properties. The importance of corticostriatal interactions has been demonstrated across diverse contexts, ranging from simple reinforcement learning decisions^28^ to violations in trust^29^. In the case of affective and informative reward properties, differential PPI effects with VLPFC—like all PPI effects—could be interpreted in two ways. First, our results could be due to affective and informative differences in context-specific (i.e., increased feedback) contributions from the striatum to the VLPFC indirectly through circuits such as the thalamus or midbrain^30^. Second, our results could reflect affective and informative differences in how the striatum modulates VLPFC responses to increasing feedback. These divergent interpretations apply to all PPI studies^21^. Yet, we also note that our findings could also reflect a regulatory^31^ or a control^32^ mechanism in which VLPFC modulates activation within the striatum. Disambiguating these disparate accounts of our connectivity findings would require alternative analytical approaches such as dynamic causal modeling^33^ likely coupled with improved (i.e., faster) imaging protocols^34^. Nevertheless, our results advance a new circuit-level understanding of how distinct reward properties are processed through corticostriatal interactions.

Models of brain function that explicitly leverage the fact that no brain region works in isolation are rapidly gaining traction across a wide range of domains—from perceptual^35^ to social neuroscience^36^. Circuit-level approaches may be especially important in clinical neuroscience as researchers begin to use connectivity patterns to gain insight into psychopathology^37^. Applying our circuit-level understanding of affective and informative reward properties may help refine models of psychopathologies that rely on general reward-processing deficits indexed by blunted striatal responses to reward^8^. We speculate that disorders showing similar striatal deficits could require distinct treatment strategies. For example, our results suggest that enhancing affective connectivity with VLPFC may rescue striatal responses in depressed patients^9^. Similarly, suppressing informative connectivity between the striatum and VLPFC regions may rescue striatal responses in schizophrenic patients^38^.

Although parsing reward into distinct properties carries many exciting applications, we note that our approach carries some intrinsic limitations. In particular, our procedures may not permit a complete dissociation of affective and informative properties of reward. While each of our card tasks emphasized a specific property, it is possible that our feedback stimuli were not solely affective or informative—a feature common to the experience of reward^2^. We were able to demonstrate a selective relationship between NAcc responses to informative properties and subsequent utilization of information, but we were unable to report an analogous finding for affective properties. Despite our efforts to try to emphasize affective and informative properties in distinct card games, we cannot rule out that both card tasks modulated evoked affect, given our difficulty of quantifying online affective responses. Future work will have to build on our findings with improved quantification of online affective responses, potentially through pupillometry^39^, heart rate^40^, and other measures that provide insight into the affective nature of reward at the time of experience. In addition, while both of our tasks involved working memory, it is also worth noting that working memory was more explicit in the ICT due to its structure and relationship with subsequent payment. Nevertheless, we emphasize that information is only valuable insofar as it can be remembered and applied later. Disentangling working memory and informative reward properties may be an intractable problem, but selective disruptions of working memory could provide interesting insight into the mechanisms underlying informative reward properties^41^.

Other task and behavioral differences may have also contributed to our findings. For instance, although behavioral differences between the tasks (i.e., preferences and choice persistence) were generally uncorrelated with neural differences, it is possible that participants’ overall preference for the affective task may have boosted attention, thus amplifying neural responses to affective feedback^42^. The immediacy between action and feedback may have also differed between the two tasks. Although we controlled for differences between action and reward (money) with our bonus task structure, we note that the feedback necessary to eventually receive the reward was different between the two tasks. Specially, the ACT used feedback (points) that was more concrete and immediately useful while the ICT used feedback (bits) that was less concrete and only useful in the future.

These limitations may have precluded our ability to observe dissociable signals within the striatum. Prior work has suggested that dorsal and ventral striatum may have distinct roles in reward processing. For example, dorsal striatum responds to information (e.g., learning action-outcome associations)^43^ while the ventral striatum responds to affective cues (e.g., sexual imagery)^13^. These observations suggest that affective and informative reward properties may be encoded in ventral and dorsal striatum, respectively; however, we did not observe this dissociation within our study. Although the absence of an effect cannot be interpreted, we speculate that the structure of our procedures—particularly the delayed receipt of monetary reward—may have attenuated action-outcome signals in the dorsal striatum. Alternatively, striatal signals that decode affective and informative properties could be imbedded within high-frequency spatial patterns, which would necessitate the application of multivariate pattern analysis to high-resolution fMRI data^44^. Taken together, future work aiming to segregate responses within the striatum may need to employ alternative task structures combined with improved imaging and analytic strategies.

In summary, our work provides an important step toward identifying the underlying mechanisms that support affective and informative properties of reward. Illuminating how these properties differentially impact behavior and brain function could have immediate impact on models of psychopathology^8^ and learning strategies within educational systems^11^. Moreover, we speculate that affective and informative properties are imbedded within social signals, such as feedback received from another person^45^,^46^,^47^. Understanding how affective and informative properties shape our social interactions has the potential to redefine our approach to disorders marked by aberrant social processing, including anorexia nervosa^48^ and autism^49^,^50^.

## Methods

### Participants

Thirty-three individuals (18 females) participated in the neuroimaging study (mean age: 24 years, range: 18–39 years). Prescreening excluded individuals with a history of psychiatric or neurological illness. All procedures and methods were conducted in accordance with guidelines approved by the Institutional Review Board at Rutgers University. Written informed consent was obtained from all participants.

### Stimuli and Tasks

In the scanner, affective and informative reward properties were probed using two card-guessing tasks presented in an interleaved fashion during the experiment. The card tasks shared an identical trial structure but emphasized distinct goals: the *Affective Card Task* (ACT; Fig. 1A) required participants to acquire as many points as possible whereas the *Informative Card Task* (ICT; Fig. 1B) required participants to acquire information regarding the decks of cards. Although these goals may introduce differences in how participants approached the task, we note that each goal had direct implications for final payment to the participant: earning enough points permits entry into a bonus game for additional monetary compensation; but, performing well in the bonus game requires using information learned in the ICT. Thus, while each card task delivers feedback that contains affective and informative reward properties, we stress that each task emphasizes the intended reward property within the context of a bonus game delivering monetary compensation. Notably, this general structure helps mitigate differences in the immediacy of affective and informative reward properties because the actual reward (money) is not received until the end of the experiment and depends on meeting the goals of both tasks.

Participants completed two 36-trial runs of each card task; these runs were interleaved and with order counterbalanced across participants. On each trial, participants were presented with three decks of cards for up to 2.5 seconds or until selecting a deck. After a variable fixation interval of 3.25–6.25 seconds, a card (feedback) from the chosen deck was presented for 1 second. Failure to respond within the required interval resulted in no feedback, which was shown as a black circle. In the ACT, cards depicted variable amount of points (1, 2, 3) appearing with different probabilities in each deck (50%, 33%, 17%). The point distributions within each deck were as follows: Deck 1: 1 = 50%, 2 = 33%, 3 = 17%; Deck 2: 1 = 17%, 2 = 50%, 3 = 33%; Deck 3: 1 = 33%, 2 = 17%, 3 = 50%. This task structure, while imbuing some elements of information within the ACT, ensures participant engagement while also helping to equate the ACT and ICT in terms of working memory demands. Similarly, in the ICT, cards depicted letters (D, K, X) appearing with different probabilities in each deck (50%, 33%, 17%). Although these probabilities reflect the average probabilities that a given letter will be drawn from a specific deck, we note that subjective probabilities experienced by the participant change based on accumulated evidence (see Supplemental Methods). We therefore assumed that participants started the ICT with flat expectations that were updated on a trial-to-trial basis. We capitalized on this feature using an information-theoretic framework in which each outcome from the ICT was expressed in units of bits, where bits = −log_2_(*p*) (see Supplemental Methods). To facilitate comparisons across the ACT and ICT, each task delivered no feedback on 33% of trials, irrespective of card drawn from the deck. Trials were separated by a variable intertrial interval (ITI) of 5–11 seconds.

Following the card tasks, participants were told they earned enough points to play in the bonus game; however, before playing in the bonus game, we gave each participant an opportunity to either acquire more information or more points using a simple preference task. Here, more points equated to larger bonus (0 or 1 point: $20; 2 points: $25; 3 points: $30), allowing us to assess individual differences in subjective value for information^51^. On each trial of the *Preference Task*, participants were given up to 2.5 s to choose which of two decks they would prefer to receive a card. The two decks could come from the same card task (Forced Choice; 12 trials) or from different card tasks (Free Choice; 18 trials). Our analysis focused on the proportion of trials participants chose information during the Free Choice condition. Trials were separated by a variable ITI of 5.5 s–13.5 s. A randomly-chosen trial was resolved at the conclusion of the task. Participants also completed subjective ratings (1–7 Likert scale) on three dimensions—enjoyment, excitement, and motivation—for each of the two card tasks. All tasks were programmed with the Psychophysics Toolbox 3 in MATLAB. At the end of the experiment, all participants played the bonus game to receive monetary compensation (see Supplemental Methods) and were then debriefed.

### Neuroimaging Data Collection and Preprocessing

We used standard sequences on a 3T Siemens scanner to collect our neuroimaging data (voxel size = 3 mm^3^; repetition time = 2 seconds; see Supplemental Methods). These data were preprocessed using tools from SPM12^52^. We corrected for head motion by realigning each time series to its first volume. As part of our motion correction routines, we also applied spatial unwarping to ameliorate geometric distortions arising from susceptibility artifacts. The mean functional image was coregistered to the anatomical scan, and unified segmentation normalization of the anatomical was computed^53^ and used to reslice the functional data to standard stereotaxic space (3 mm isotropic) defined by the Montreal Neurological Institute (MNI). Finally, the normalized functional images were spatially smoothed with a 4 mm full-width-half-maximum Gaussian kernel. We also employed additional preprocessing steps to reduce the impact of head motion on connectivity analyses (see Supplemental Methods).

### Neuroimaging Analyses

We used FSL^54^ to evaluate two primary sets of models to quantify how brain regions respond to affective and informative feedback. Both sets of models utilized a general linear model (GLM) with autocorrelation correction. First, our *parametric* models identified brain regions whose activation increases as a function of increasing feedback magnitude. Each first-level GLM consisted of three regressors modeling the choice phase; these regressors explicitly controlled for response time differences within and across tasks by setting the duration of the each choice equal to response time. To model feedback-related responses, we included two additional regressors corresponding to the presentation of feedback (duration = 1 s): a constant term (0^th^ order) and a parametric term (1^st^ order) modulated by the normalized feedback magnitude (none, low, medium, high). The parametric term identified regions whose response increases (or decreases) with greater feedback magnitude. In a descriptive *post-hoc* analysis, we estimated a categorical model where feedback magnitude response levels were modeled separately.

Second, our *psychophysiological interaction (PPI)* models^21^ identified brain regions whose coupling with the striatum increased or decreased as a function of feedback magnitude (none, low, medium, high). (We note that these parametric relationships in connectivity need not be monotonically increasing or decreasing: the key consideration is the slope of the line going through the four levels of feedback magnitude). We first evaluated two separate PPI models, each relating to a discrete functional region of interest (ROI) in the striatum: 1) a striatal region linked to the conjunction of affective and informative responses [right nucleus accumbens; MNI_x,y,z_ = 9, 14, −7 (26 voxels)]; and 2) a striatal region linked to the difference of affective and informative responses [left ventral striatum; MNI_x,y,z_ = −12, 5, −13 (22 voxels)]. Both PPI models utilized the same regressors as the parametric model, but also included a physiological regressor representing the time course of activation within the striatal ROI under consideration. To form the PPI regressor in each model, we multiplied the (convolved) physiological regressor of interest by the (convolved) regressor modeling the normalized participant-specific feedback magnitude (i.e., the 1^st^-order parametric term from our parametric model).

Given the complex, overlapping pattern of inputs to the striatum^22^,^23^, we conducted an additional connectivity analysis involving independent component analysis (ICA), which has been validated in previous work^16^. Briefly, we submitted functional data from the striatum (defined by the union of the caudate, putamen, and nucleus accumbens from the Harvard-Oxford atlas) to a group ICA, which identified 10 independent striatal networks across participants. The group-level striatal networks were then regressed onto the functional data to recover participant-specific responses for each striatal network^16^. We identified one striatal network that encoded both affective and informative reward properties; the time course of activation for this task-sensitive striatal network was then used as the physiological regressor in a PPI model. To accurately capture striatal ICA maps in individual subjects^55^,^56^, this model also included the responses of other striatal networks that did not respond to the task but was otherwise identical to the PPI models described above. The key advance of this approach—which integrates ICA and PPI—is the ability to quantify coupling with distributed, yet overlapping, response patterns in the striatum^15^,^16^,^47^.

In both sets of models, we included nuisance regressors to account for missed responses and temporal derivatives for task regressors. Inclusion of temporal derivatives helps mitigate potential differences in HRF latencies across tasks, though only up to approximately one second^57^. All task regressors were convolved with the canonical hemodynamic response function. We combined data across runs, for each participant, using a fixed-effects model that included a covariate for the proportion of motion spikes in each run; this participant-level model allowed us to contrast the ACT and ICT while controlling for inter-session differences in head motion. Finally, we combined data across participants using a mixed-effects model^58^. Except where noted, all *z*-statistic images were thresholded and corrected for multiple comparisons using an initial cluster-forming threshold of *z* > 3.1 followed by a conservative whole-brain corrected cluster-extent threshold of *p* < 0.05, as determined by Gaussian Random Field Theory^59^,^60^.

## Additional Information

**How to cite this article**: Smith, D. V. *et al.* Distinct Reward Properties are Encoded via Corticostriatal Interactions. *Sci. Rep.* **6**, 20093; doi: 10.1038/srep20093 (2016).

## Supplementary Material

Supplementary Information

## Figures and Tables

**Table 1:** Regions Encoding Affective Reward Properties.

Probabilistic Anatomical Label	Peak *Z*-stat	x	y	z	Cluster Size	P-value (corrected)
Occipital Pole (62%)	6.76	−27	−97	−10	506	4.00E-12
Occipital Pole (49%), iLOC (12%)	6.04	27	−94	−10	409	1.84E-10
MFG (48%), IFGpt (8%)	4.96	48	29	29	393	3.56E-10
SPL (31%), pSMG (30%), Angular Gyrus (6%)	5.17	−42	−46	50	341	3.24E-09
sLOC (60%)	4.8	30	−64	44	307	1.46E-08
IFGpo (34%), Precentral Gyrus (17%), MFG (9%)	4.86	−45	11	26	307	1.46E-08
Paracingulate Gyrus (74%), ACC (10%)	4.95	0	38	29	293	2.75E-08
Left Accumbens (87%)	5.31	−9	8	−7	216	1.13E-06
Frontal Pole (26%)	4.97	−39	41	2	183	6.20E-06
Right Accumbens (81%)	5.02	9	14	−7	159	2.30E-05
PCC (45%)	4.75	−3	−25	26	150	3.83E-05
Frontal Orbital Cortex (58%)	4.43	36	26	−10	70	0.00642

Clusters whose activation increases with increasing affective reward properties. Probabilistic labels reflect the probability (or likelihood) that a coordinate belongs to a given region. For clarity, we only show labels whose likelihood exceeds 5%. Abbreviations: iLOC (lateral occipital cortex, inferior division); SPL (superior parietal lobule); pSMG (supramarginal gyrus, posterior division); MFG (middle frontal gyrus); IFGpo (inferior frontal gyrus, pars opercularis); PCC (cingulate gyrus, posterior division); ACC (cingulate gyrus, anterior division).

**Table 2:** Regions Encoding Informative Reward Properties.

Probabilistic Anatomical Label	Peak Z-stat	x	y	z	Cluster Size	P-value (corrected)
SPL (10%), Angular Gyrus (8%)	5.07	−33	−52	35	321	4.88E-12
IFGpo (51%)	5.25	−51	17	23	269	1.25E-10
sLOC (32%), SPL (15%), Angular Gyrus (10%)	5.4	33	−58	44	237	1.03E-09
Occipital Pole (53%), iLOC (10%)	6.35	−27	−94	−10	196	1.75E-08
Precentral Gyrus (24%), MFG (21%), IFGpo (15%)	4.88	39	8	32	161	2.38E-07
Occipital Pole (47%), iLOC (23%)	5.02	33	−91	−10	159	2.98E-07
Paracingulate Gyrus (47%), ACC (31%)	4.59	−6	14	41	118	7.33E-06
MFG (31%), Precentral Gyrus (26%)	4.22	−36	−1	56	54	0.00322
Insular Cortex (63%), Frontal Orbital Cortex (8%)	4.7	36	20	−4	54	0.00322
Right Accumbens (81%)	4.23	9	14	−7	38	0.0205
Insular Cortex (45%), Frontal Orbital Cortex (28%)	4.56	−33	20	−7	32	0.0434

Clusters whose activation increases with increasing informative reward properties. Probabilistic labels reflect the probability (or likelihood) that a coordinate belongs to a given region. For clarity, we only show labels whose likelihood exceeds 5%. Abbreviations: MFG (middle frontal gyrus); IFGpo (inferior frontal gyrus, pars opercularis); iLOC (lateral occipital cortex, inferior division); sLOC (lateral occipital cortex, superior division); SPL (superior parietal lobule).

**Table 3:** Regions Encoding Affective and Informative Reward Properties.

Probabilistic Anatomical Label	Peak Z-stat	x	y	z	Cluster Size	P-value (corrected)
Occipital Pole (53%), iLOC (10%)	6.35	−27	−94	−10	162	4.52E-13
Occipital Pole (47%), iLOC (23%)	5.02	33	−91	−10	154	1.33E-12
SPL (43%), pSMG (23%), Angular Gyrus (10%)	4.12	−39	−49	50	127	5.81E-11
sLOC (60%)	4.26	30	−64	44	86	5.96E-08
IFGpo (38%), MFG (14%), Precentral Gyrus (11%)	4.46	−45	14	26	83	5.96E-08
MFG (37%), IFGpt (19%), IFGpo (9%)	4.01	51	26	26	26	0.00379
**Right Accumbens (81%)**	**4.23**	**9**	**14**	**−7**	**26**	**0.00379**
Frontal Orbital Cortex (48%), Insular Cortex (24%)	4.28	−30	23	−7	25	0.00485
Insular Cortex (51%), Frontal Orbital Cortex (19%)	3.97	39	20	−4	24	0.00623
Precentral Gyrus (32%), IFGpo (26%), MFG (5%)	3.92	48	11	29	21	0.0135
Paracingulate Gyrus (75%), ACC (7%), SFG (5%)	4.00	3	23	44	17	0.0396
pSMG (32%)	3.72	48	−37	38	17	0.0396

Clusters surviving a conjunction analysis of both reward properties. Our PPI analysis utilized the cluster within the right accumbens (bolded). Probabilistic labels reflect the probability (or likelihood) that a coordinate belongs to a given region. For clarity, we only show labels whose likelihood exceeds 5%. Abbreviations: MFG (middle frontal gyrus); IFGpo (inferior frontal gyrus, pars opercularis); iLOC (lateral occipital cortex, inferior division); sLOC (lateral occipital cortex, superior division); SPL (superior parietal lobule); SFG (superior frontal gyrus); ACC (cingulate gyrus, anterior division); pSMG (supramarginal gyrus, posterior division); IFGpt (inferior frontal gyrus, pars triangularis).
