# Supplementary material for: Distinct Reward Properties are Encoded via Corticostriatal Interactions
Source: Sci Rep. 2016 Feb 2;6:20093. doi: 10.1038/srep20093 (PMC4735713; doi:10.1038/srep20093)
Supplement: Supplementary Information [file srep20093-s1.pdf]

Supplementary Information

Distinct Reward Properties are Encoded via Corticostriatal Interactions

David V. Smith, Anastasia E. Rigney, Mauricio R. Delgado

Department of Psychology, Rutgers University, Newark, NJ 07102, USA

Supplemental Methods

Bonus Task and Monetary Compensation

At the end of the session, participants completed the bonus game to win money. This game comprised 9 trials requiring the participant to use information learned in the ICT. On each trial, a letter was shown flanked by two decks from the ICT. The participant was asked to report which deck was most likely to yield the displayed letter. The proportion of correct responses (M = 69.36%; SE = 3.45%) determined monetary compensation. In total, we paid participants a base rate of $30 plus their earnings in the bonus game, yielding a mean payment of $47.70.

Behavioral Analyses

We used MATLAB R2014a (The MathWorks, Inc., Natick, MA) and Stata 13.1 (StataCorp, College Station, TX) for behavioral analyses. One of our analyses focused on characterizing the magnitude of feedback in the ICT. Although the concept of feedback is clear within the ACT (i.e., variable levels of points), a similar construct is not readily apparent within the ICT, which presents letters that occur with varying probabilities. Given the structure of the ICT, however, we utilized a classic information-theoretic approach to estimate the amount of information conveyed by the receipt of a given letter in the ICT [1]. Here, the information of a received outcome—expressed in units of bits, where bits = -$\log_2(p)$—is based on the subjective probability of that outcome occurring given past

experience. The number of occurrences of an event (e.g., drawing a K from Deck 1) relative to other possible events (e.g., drawing a D or X from Deck 1) determines the subjective probability p of that event; for example, $p = N_K / (N_D + N_K + N_X)$. Because no events have occurred prior to the first trial, we assume the expectations are flat for each event (N = 1, which yields p = 33%). If the participant draws a K from Deck 1 on the first trial, then the history of Deck 1 is updated simply through counting ($N_D = 1$; $N_K = 2$; $N_X = 1$). Drawing another K from Deck 1 on the second trial would therefore be less informative (bits = $-\log_2(2/(1+2+1))$) than drawing a D or X from Deck 1 (bits = $-\log_2(1/(1+2+1))$). Thus, an event that has occurred with regularity (high p) carries fewer bits than an event that has not occurred with regularity (low p). We examined the behavioral effects of points (affective reward properties) and bits (informative reward properties) using a two (feedback type: affective or informative) by three (magnitude: low, medium, high) repeated measures analysis of variance. This analysis excluded the "No Feedback" condition because our key question here is, given feedback, how does the feedback magnitude influence choice. (We note that we included the no feedback condition within our neuroimaging analyses to facilitate comparisons of parametric responses across both tasks [2].) All reported p-values have been adjusted (using the Huynh-Feldt episilon) to account for violations of sphericity.

Skin Conductance Recordings and Analyses

We recorded galvanic skin responses using AcqKnowledge software (BIOPAC Systems Inc). Isotonic gel electrodes were attached to the intermediate phalanges on the first and second digits of the left hand. Data were recorded using a sampling rate of 200 Hz. We used Ledalab and MATLAB to process and analyze the data. Raw data were downsampled by a factor of 20 and smoothed with a kernel of 10 samples to attenuate noise resulting from concurrent fMRI scanning. We quantified event-locked skin conductance responses (SCRs) as the trough-to-peak amplitude difference of the largest response within a window spanning 0.5 s to 5 s after the onset of feedback. We used a minimal response criterion of 0.001 μS; responses below this threshold were scored as "0". Prior to analysis, SCRs were square-root transformed to reduce skewness and were z-transformed within subjects to facilitate comparisons across subjects. In addition, we also

excluded subjects who failed to reliably show task-evoked GSR responses (i.e., a minimum of a 30% response rate across trials). This criterion identified 14 subjects who could be included in the final SCR analyses.

Neuroimaging Data Acquisition

Neuroimaging data were collected using a 3T Siemens MAGNETOM Trio scanner (equipped with 12 channels) at the Rutgers University Brain Imaging Center (RUBIC). Functional images sensitive to blood-oxygenation-level-dependent (BOLD) contrast were acquired using a single-shot T_2*-weighted echo-planar imaging sequence with slices parallel to the axial plane [GRAPPA with R = 2; repetition time (TR): 2000 ms; echo time (TE): 30 ms; matrix 68 x 68; field of view (FOV): 204 mm; voxel size 3.0 x 3.0 x 3.0 mm; 37 slices (10% gap); flip angle: 90°]. We also collected B_0 field maps (TR: 402 ms; TE_1: 7.65 ms; TE_2: 5.19 ms; flip angle: 60°) using the same slice prescription and voxel dimensions as the functional images. High-resolution structural scans covering the whole brain (TR: 1900 ms; TE: 2.52 ms; matrix 256 x 256; FOV: 256 mm; voxel size 1.0 x 1.0 x 1.0 mm; 176 slices; flip angle: 9°) were acquired to facilitate coregistration and normalization of functional data.

Additional Controls for Head Motion

Given that brain connectivity results can be severely distorted by head motion, we applied additional corrections and controls for head motion that are commonly used by other groups (for review, [3]). These additional preprocessing steps were carried out using tools from FSL (FMRIB Software Library version 5.0.4; http://www.fmrib.ox.ac.uk/fsl) [4]. First, we identified motion spikes using an FSL tool called *fsl_motion_outliers*. We used two metrics for assessing motion spikes: 1) root-mean-square (RMS) intensity difference of each volume relative to a reference volume (the first time point); and 2) frame-wise displacements computed as the mean RMS change in rotation/translation parameters relative to a reference volume (the first time point). For each metric, we used a boxplot threshold (i.e., 75th percentile plus 1.5 times the interquartile range) applied to the metric values within a run to classify volumes as spikes. All spikes were then removed via regression [3,5]. This procedure removed an average of 7.6% of volumes (range: 0 to 16%).

Importantly, following removal of motion spikes, no subjects exhibited extreme average volume-to-volume head motion (M = 0.047 mm; range: 0.021 to 0.081 mm) or maximum volume-to-volume head motion (M = 0.118 mm; range: 0.045 to 0.367 mm). Second, non-brain material was removed from the functional images [6], and the entire 4D dataset was grand-mean intensity normalized using a single multiplicative factor. Finally, to remove low frequency drift in the MR signal, we used a high-pass temporal filter with a 100 second cutoff (Gaussian-weighted least-squares straight line fitting, with sigma = 50 s). Notably, applying the temporal filter after removing motion spikes minimizes ringing artifacts [5,7,8].

We further preprocessed our data by filtering out artifacts identified in an independent component analysis (ICA) [9]. For each dataset, we conducted a probabilistic independent component analysis [10] as implemented in MELODIC (Multivariate Exploratory Linear Decomposition into Independent Components) Version 3.10, part of the FSL software package. Prior to estimating each ICA, input data were demeaned and the variance was normalized across voxels. The number of dimensions was estimated using the Laplace approximation to the Bayesian evidence of the model order [10]. The whitened observations were then decomposed into sets of vectors that describe signal variation across the temporal domain (time-courses) and across the spatial domain (maps) by optimizing for non-Gaussian spatial source distributions using a fixed-point iteration technique [11]. We normalized the estimated component maps by dividing the maps by the standard deviation of the residual noise. Normalized component maps were then submitted to a classifier to automatically label components as signal and noise [9]. Independent components labeled as noise were then filtered from each dataset using regression [9]. Finally, we also regressed out residual variance tied to motion parameters and extended motion parameters (i.e., squares, temporal differences, and squared temporal differences) [3]. Taken together, these additional controls for head motion are helpful in mitigating concerns that our connectivity results are influenced by subtle differences in head motion [3].

Convolution within the Psychophysiological Interaction Analyses

To form the PPI regressor in each model, we multiplied the (convolved) physiological regressor of interest by the (convolved) regressor modeling the normalized participant-specific feedback magnitude (i.e., the 1st-order parametric term from our

parametric model). Although it has been argued that it is important to form the PPI regressor using deconvolved regressors [12], we note that our approach—which is typical within the FSL package [13]—assumes that the shape of the hemodynamic response function is comparable for the task and physiological regressors.

Statistical Thresholding

Except where noted, all z-statistic images were thresholded and corrected for multiple comparisons using an initial cluster-forming threshold of $z > 3.1$ followed by a corrected cluster-extent threshold of $p < 0.05$ [14]. Statistical overlay images were created using MRIcroN and MRIcroGL. As Brodmann labels do not depict anatomical variation [15], we show probabilistic anatomical labels for activation maxima using the Harvard-Oxford cortical and subcortical atlases.

References

1 Baldi, P. & Itti, L. Of bits and wows: A Bayesian theory of surprise with applications to attention. *Neural networks : the official journal of the International Neural Network Society* **23**, 649-666, doi:10.1016/j.neunet.2009.12.007 (2010).

2 Buchel, C., Holmes, A. P., Rees, G. & Friston, K. J. Characterizing Stimulus-Response Functions Using Nonlinear Regressors in Parametric fMRI Experiments. *Neuroimage* **8**, 140-148 (1998).

3 Power, J. D., Schlaggar, B. L. & Petersen, S. E. Recent progress and outstanding issues in motion correction in resting state fMRI. *Neuroimage* **105C**, 536-551, doi:10.1016/j.neuroimage.2014.10.044 (2015).

4 Smith, S. M. *et al.* Advances in functional and structural MR image analysis and implementation as FSL. *Neuroimage* **23 Suppl 1**, S208-219, doi:10.1016/j.neuroimage.2004.07.051 (2004).

5 Satterthwaite, T. D. *et al.* An improved framework for confound regression and filtering for control of motion artifact in the preprocessing of resting-state functional connectivity data. *Neuroimage* **64**, 240-256, doi:10.1016/j.neuroimage.2012.08.052 (2013).

6 Smith, S. M. Fast robust automated brain extraction. *Hum. Brain Mapp.* **17**, 143-155, doi:10.1002/hbm.10062 (2002).

7 Carp, J. Optimizing the order of operations for movement scrubbing: Comment on Power et al. *Neuroimage* **76**, 436-438, doi:10.1016/j.neuroimage.2011.12.061 (2013).

8 Weissenbacher, A. *et al.* Correlations and anticorrelations in resting-state functional connectivity MRI: A quantitative comparison of preprocessing strategies. *Neuroimage* **47**, 1408-1416 (2009).

9 Salimi-Khorshidi, G. *et al.* Automatic denoising of functional MRI data: combining independent component analysis and hierarchical fusion of classifiers. *Neuroimage* **90**, 449-468, doi:10.1016/j.neuroimage.2013.11.046 (2014).

10 Beckmann, C. F. & Smith, S. M. Probabilistic independent component analysis for functional magnetic resonance imaging. *IEEE Trans. Med. Imaging* **23**, 137-152 (2004).

11 Hyvarinen, A. Fast and robust fixed-point algorithms for independent component analysis. *IEEE Trans Neural Netw* **10**, 626-634, doi:10.1109/72.761722 (1999).

12 Gitelman, D. R., Penny, W. D., Ashburner, J. & Friston, K. J. Modeling regional and psychophysiologic interactions in fMRI: the importance of hemodynamic deconvolution. *Neuroimage* **19**, 200-207 (2003).

13 O'Reilly, J. X., Woolrich, M. W., Behrens, T. E., Smith, S. M. & Johansen-Berg, H. Tools of the trade: psychophysiological interactions and functional connectivity. *Soc. Cogn. Affect. Neurosci.* **7**, 604-609, doi:10.1093/scan/nss055 (2012).

14 Worsley, K. J. in *Functional MRI: An Introduction to Methods* (eds P. Jezzard, P.M. Matthews, & S.M. Smith) Ch. 14, (Oxford University Press, 2001).

15 Zilles, K. & Amunts, K. Centenary of Brodmann's map--conception and fate. *Nat. Rev. Neurosci.* **11**, 139-145, doi:10.1038/nrn2776 (2010).
